# Relationship between Porcine Sperm Motility and Sperm Enzymatic Activity using Paper-based Devices

**DOI:** 10.1038/srep46213

**Published:** 2017-04-07

**Authors:** Koji Matsuura, Han-Wei Huang, Ming-Cheng Chen, Yu Chen, Chao-Min Cheng

**Affiliations:** 1 Department of Biomedical Engineering, Faculty of Engineering, Okayama University of Science, 1-1 Ridai-Cho Kita-Ku, Okayama, 700-0005, Japan; 2 Institute of Biomedical Engineering, National Tsing Hua University, Hsinchu 30013, Taiwan; 3 Department of Urology, Chang Gung Memorial Hospital and Chang Gung University, Taoyuan 33302, Taiwan

## Abstract

Mammalian sperm motility has traditionally been analyzed to determine fertility using computer-assisted semen analysis (CASA) systems. To develop low-cost and robust male fertility diagnostics, we created a paper-based MTT assay and used it to estimate motile sperm concentration. When porcine sperm motility was inhibited using sperm enzyme inhibitors for sperm enzymes related to mitochondrial activity and glycolysis, we simultaneously recorded sperm motility and enzymatic reactivity using a portable motility analysis system (iSperm) and a paper-based MTT assay, respectively. When using our paper-based MTT-assay, we calculated the area mean value signal intensity (AMV) to evaluate enzymatic reactivity. Both sperm motility and AMV decreased following treatment with iodoacetamide (IODO) and 3-bromopyruvic acid (3BP), both of which are inhibitors of glycolytic enzymes including glyceraldehyde-3-phosphate dehydrogenase (GAPDH). We found a correlation between recorded motility using iSperm and AMV from our paper-based assay (P < 0.05), suggesting that a sperm-related enzymatic reaction is involved in sperm motility. Under this protocol, MTT reduction was coupled with catalysis of GAPDH and was promoted by electron transfer from NADH. Based on this inhibitor study, sperm motility can be estimated using our paper-based MTT-assay.

Sperm motility and sperm motion velocities can be tracked using computer-assisted semen analysis (CASA) an approach that refers to a variety of automatic or semi-automatic, often image-tracking, approaches to indicate fertility in analyzed semen. This approach has been used to examine fish sperm in water samples^1^, to assist in animal husbandry^2^,^3^, for human assisted reproductive technology (ART)^4^,^5^,^6^, and is a frequently used tool for pre-analysis prior to artificial insemination (AI) attempts^2^,^3^. Some research results present significant correlations between sperm motility and fertility for cattle^3^. CASA results can show the percentage of motile sperm, linear velocity, curvilinear velocity, and head frequency based on trajectories of motile sperm. Introducing CASA in an AI center is sometimes difficult in resource-limited settings because conventional CASA systems usually require optical microscopic system and software including trajectory analysis algorithms. A simple but robust sperm motility analysis system that could be used for cattle breeding centers in developing countries would reduce the cost of sperm motility analyses.

Recent smartphone-based technologies may overcome this problem and lead to the development of alternative sperm motility analysis systems^7^,^8^. For example, smartphone-based CASA applications are currently commercially available; they include iSperm, which functions on the Apple iOS^8^. An iSperm system requires no more than a smartphone with a lens and the iSperm application. Using this system, one can evaluate the percentage of motile sperm (sperm motility), but not motile sperm trajectory information. Using this portable system, sperm motility can be evaluated in rural, resource-limited settings. High linear velocity sperm and progressive motility analyzed using CASA are good indicators for fertility prediction^9^,^10^,^11^,^12^. Despite the shortcomings of iSperm, i.e, it cannot record sperm motion trajectories, higher cost performance was obtained using iSperm (1,000 USD) in these settings compared with the initial cost of CASA, which is usually approximately 10,000 USD.

CASA and a smartphone-based motility analysis system can be used to determine sperm trajectory and motion under optical microscopic observation^13^,^14^,^15^. Because mammalian sperm motility is expressed by motor protein function using adenosine triphosphate (ATP) catalysis, ATP shuttle system, and ATP production system^16^,^17^, analyses of sperm cell chemical reactions may also be used as potential indicators of sperm motility^17^. We have developed a paper-based motility analysis system that relies on reduction of 3-(4,5-dimethyl thiazol-2-yl)-2,5-diphenyl tetrazolium salt (MTT)^18^,^19^. Our paper-based MTT assay experimental results suggest that MTT color intensity changes by MTT reduction could be correlated to sperm motility, so this system may be applicable for fertility examinations^18^,^19^. These results were consistent with other research groups reporting on the use of paper-based MTT assays for human sperm motility^20^. Physiological functions, especially redox chemistry with exogenous compounds such as MTT, are not well discussed regarding porcine sperm to date and additional investigation of this sperm detection technology is required for implementation in cattle breeding. We initially needed to explain paper-based device functionality. To explain functionality based on molecular mechanisms of sperm and chemical sperm motility inhibition is also required; however, we did not prepare a complete inhibitor study for porcine sperm, electing to examine inhibition by studying physiological insights of porcine sperm before use of our clinical sample.

While we have made advances in this technology for clinical application, to determine more complete effectiveness of our system we must demonstrate the relationship between MTT reduction and physiological functions related to sperm motility. To find the clues to this relationship, we must first touch on mammalian sperm ATP production mechanisms, as they provide energy for sperm motility. Sperm ATP is produced by an electron transfer system in mitochondria and a glycolysis system in flagellum cytosol, and the origin of ATP for mammalian sperm motility is currently under debate^20^,^21^,^22^. Recent studies report that ATP produced by glycolysis played a key role in mouse sperm motility based on knock-out mice or chemical inhibition studies^23^,^24^. An ATP-transferring mechanism from mitochondria to distal flagellum was also proposed, and glycolytic enzymes such as glyceraldehyde-3-phosphate dehydrogenase (GAPDH) were involved in this mechanism^22^,^23^. Because MTT reduction must be coupled with redox reaction during sperm ATP production, and this system includes redox mediator reactions such as nicotinamide adenine dinucleotide (reduced form) (NADH)^25^,^26^, the effectiveness of an MTT reduction assay for sperm motility analysis can be strengthened based on simultaneous sperm motility analysis and an MTT assay examining inhibition of mitochondrial or glycolytic enzymatic functions. Use of iSperm and a paper-based system capable of performing the above analyses would be highly effective in resource-limited settings.

Here, we investigated the effects of motility inhibitors and enzymatic reaction activity in porcine sperm to establish a relationship between MTT reduction and physiological functions related to sperm motility. Rotenone (ROT) decreases mitochondrial electron transfer^27^, specifically between NADH dehydrogenase and ubiquinone, whereas 2-deoxy-D-glucose (DOG) inhibits glucose uptake through plasma membrane glucose transporters, and iodoacetamide (IODO) reduces the reactivity of glycolysis in sperm^24^,^28^,^29^. 3-Bromopyruvic acid (3BP) inhibits both mitochondrial electron transfer (complex II) and glycolysis including the functions of hexokinase II (HK II) and GAPDH^30^,^31^,^32^,^33^,^34^,^35^. The functions of these inhibitors are shown in Table 1. Our experimental design is summarized in Fig. S1.

## Experimental Section

### Preparation of paper-based devices

**Figure 1:**
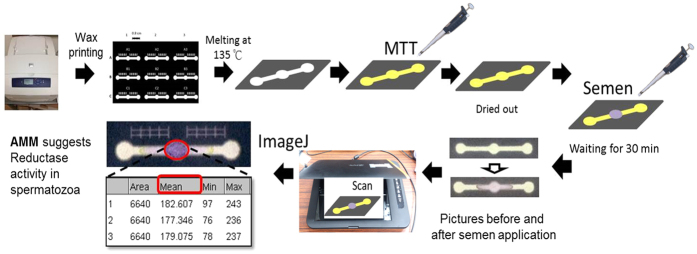
Paper-based MTT assay schematic.

We coated our paper-based device with MTT (thiazolyl blue tetrazolium bromide, ≥97.5%, Sigma-Aldrich, St. Louis, MO, USA) according to the refs 18 and 19. For this experiment, we designed a simple microfluidic pattern using Microsoft PowerPoint software (Microsoft, Redmond, WA, USA)^36^,^37^. The full length of the pattern is 2.8 mm; the circular diameter is 0.4 mm. We printed the pattern on chromatography paper (Grade 1 Qualitative Filter Papers, GE Healthcare Life Sciences, Little Chalfont, Bucks, UK), as shown in Fig. 1 using wax-printer (Phaser 8560 wax printer, Xerox, Norwalk, CT, USA)^36^,^37^. After baking the paper using the patterned wax at 105 °C for 3 min, hydrophilic-hydrophobic regions formed inside the chromatography paper. MTT aqueous solution (5 mg/mL) was added onto the hydrophilic reaction channel, and the solution was dried in the shade for 30 min.

### Porcine Sperm Preparation

Porcine sperm was supplied by the AI Center of Agricultural Technology Research-Institute Animal Technology Laboratories in Taiwan. Semen was collected and used on the same day for these experiments. The preparation of porcine sperm from the AI center briefly summarized here. First, high-quality boar (Duroc swine) semen was selected. Second, semen volume, color, and smell were analyzed. Third, semen characteristics and motility were recorded using optical microscopy. Fourth, sperm concentration, pH value, and osmolality were evaluated. After confirming qualified sperm, the analyzed semen was transported in a disinfected styrofoam box to prevent pathogen contamination during the transition to our laboratory. Before these experiments, semen was mixed gently and evenly. Upon receiving the semen in our laboratory, we unpacked it and immediately placed it into warm water, where it was maintained at a temperature of 15–18 °C.

### Sperm assay and inhibitor treatment experiments

We selected and purchased chemicals for inhibition experiments: Rotenone (Sigma-Aldrich, St. Louis, MO, USA); 2-Deoxy-D-glucose (DOG, ≥99%, Sigma-Aldrich, St. Louis, MO, USA); Iodoacetamide (IODO, ≥99%, Sigma-Aldrich, St. Louis, MO, USA); 3-bromopyruvate (3BP, 97%, Alfa Aesar, Ward Hill, MA, USA); 3-nitropropanoic acid (3NPA, ≥97%, Sigma-Aldrich, St. Louis, MO, USA); dimethyl sulfoxide (DMSO, J.T.Baker, Center Valley, PA, USA). We examined sperm metabolic activity characteristics by examining the effects of these inhibitors and prepared samples for analysis by incubating them in a water bath for 15 min at 37.5 °C. We used a hot plate to maintain their temperature. We applied different volumes of inhibitors to create a variety of inhibitor-to-sperm concentrations and mixed them together rapidly. We executed a time-course test to determine sperm motility inhibition. Following 0, 20, 40, and 60 min periods of inhibitor mixing with original semen, we tested each sample by iSperm and paper-based MTT assay simultaneously. A total of 5 μL of semen was applied to the middle circle of the paper-based device. The reaction of MTT on the paper proceeded for 20–30 min in the dark before we scanned the color-changed paper device as shown in Fig. 1. To evaluate dose-dependent effect, we added 0, 0.25, 0.5, 1 and 2 mM of IODO or 0, 0.2, 0.4, 0.6 and 0.8 mM of 3BP to porcine semen and waited for 60 min.

### Sperm Motility Analysis

We used iSperm (Aidmics biotechnology, Taipei, Taiwan) as the standard to compare against our paper-based device. iSperm consists of a balloon camera connected with an iPAD mini with sperm motility analysis software installed on the iPAD mini. A disposable microfluidic chip (Aidmics biotechnology) was loaded onto the microscopic device, and a sample (original sperm sample about 3–5 mL) was applied at the bottom of the chip to avoid touching the bottom of the cup and making bubbles. We placed a cover on the chip vertically, opened the light source of the external microscopic device, and combined it with the iPAD mini (Apple Inc., San Jose, CA). We recorded sperm activity level (%) and concentration (ten million/mL) following 10 s of analysis. We summarized the relationship between motility assessment using the iSperm system and conventional computer-assisted sperm analysis (CASA) (Hamilton Thorne Inc., Beverly, MA, USA) in our supporting information [Fig. S2]. We analyzed the data at different time points and repeated the experiments three times to increase data accuracy (N = 3).

### MTT Assay

After applying MTT onto our paper-based platform^18^,^19^, we placed 5 μL of porcine semen onto the middle circle of our paper-based device. The ensuing enzymatic reduction reaction between sperm and MTT ensued for a minimum of 15 min. The MTT assay is a colorimetric assay (purple color) for assessing cell metabolic activity. These enzymes are capable of reducing tetrazolium to insoluble formazan^18^,^25^. We captured an image of this paper following incubation, and analyzed the color image to calculate color intensity using the area mean method (AMM). The average color intensity of pixels within the circle of interest was determined using an 8-bit grayscale. The AMM approach included a summation of pixel intensities at the region of interest and the average calculation of the signal intensity across all pixels. We used ImageJ (National Institutes of Health, Bethesda, MD, USA), to collect mean color intensity value (MV) in the circle of interest. This value corresponded to average color intensity of the spot. Lower MV and a darker, more intense signal suggested higher reduction activity. We calculated area mean value (AMV) using the following formula:


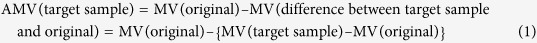


MV(original) and MV(target sample) were obtained from control sample (without inhibitors) and inhibitor-treated samples, respectively. Because calculation of AMV was easier than previous method of motility parameter (MOT) approaches, we discussed the signal intensity based on AMV^18^.

### Statistical Analyses

The sample number *N* is the number of MTT assays using five semen and three samples for drug screening analysis and dose-dependent analysis, respectively. Multiple comparisons with Dunnett’s method were chosen to evaluate significant variances between drug-treated samples at 0 min and these samples at other treatment times including dose-dependent samples. Correlation between sperm motility and the mean value from our paper-based MTT assay was determined using Pearson’s Correlation Coefficient and a corresponding significance test. Any *p*-values of <0.05 were considered statistically significant.

## Results and Discussion

### Motility analyses

**Figure 2:**
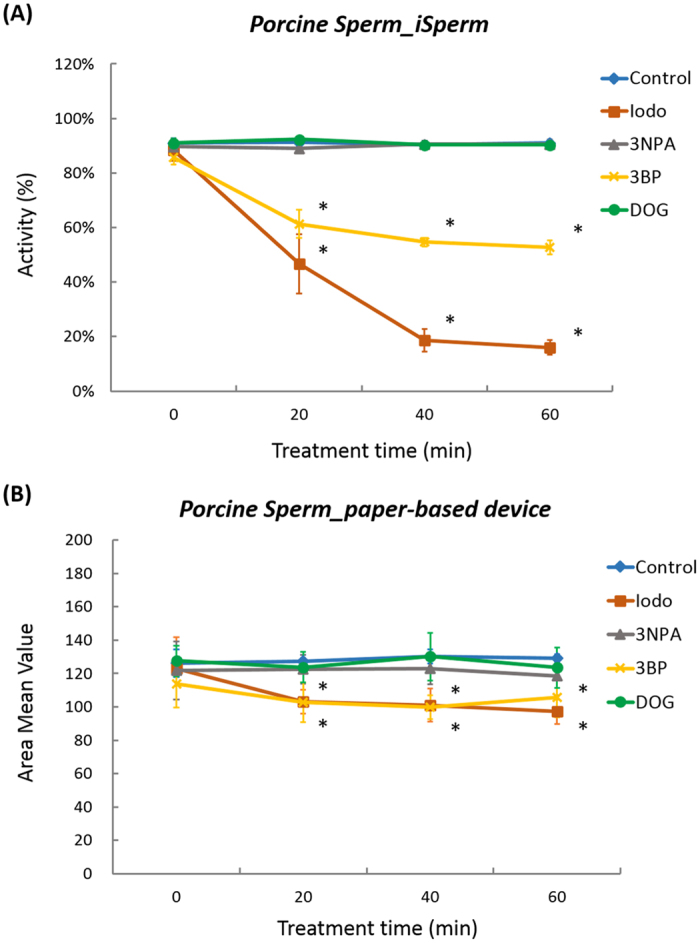
Inhibition of sperm motility recorded using iSperm and AMV of paper-based MTT assay using different inhibitors. We used (**A**) iSperm to test sperm motility and used (**B**) our paper-based device to calculate AMV. Error bars are standard deviation (SD) of three samples in each point (N = 3). Asterisks show the difference with the value of control group without inhibitor (P < 0.05). Inhibitor concentration of Iodo, 3NPA, 3BP and DOG were 2, 8, 0.2, and 8 mM, respectively.

Figure 2A shows inhibition of sperm motility using inhibitors of mitochondrial function and glycolysis. After mixing sperm with inhibitor (0 min), sperm motility of the original and the inhibitor-treated semen were approximately 90% and were quite similar, because inhibition of sperm motility had not begun. After 20, 40, and 60 min of inhibitor treatment, IODO and 3BP-treated sperm motility significantly decreased (P < 0.05). Sperm motility values from 3-NPA- and DOG-treated samples were similar to those of the control group. Although we increased concentration of the inhibitors to 8 mM, the motility was approximately 90% and almost the same as control sample motility. Treatment of porcine sperm with IODO and 3BP significantly reduced sperm motility after 20 min of incubation (P < 0.05).

### MTT Assay

Figure 2B shows the effects of inhibitor treatments on AMV using our paper-based MTT assay. After initial inhibitor mixing at 0 min (see Fig. 2B), no difference in AMV was observed between original and inhibitor-treated semen. After 20, 40, and 60 min of inhibitor treatment, we found, at most, a 30% decrease in AMV following treatment with IODO and 3BP (See Fig. S3), and determined that the AMV of the original and the IODO-treated semen were significantly different (P < 0.05). A similar trend was also observed in 3BP-treated semen samples (P < 0.05). Average AMV using 3BP after 60 min increased compared to that after 40 min. The difference of averages was less than 1 SD after 60 min. This increase is likely derived from value deviation. Treatment of porcine sperm with IODO and 3BP reduced average color intensity, suggesting a decrease in porcine sperm reductant contents.

### Inhibitor dose-dependent effects

**Figure 3:**
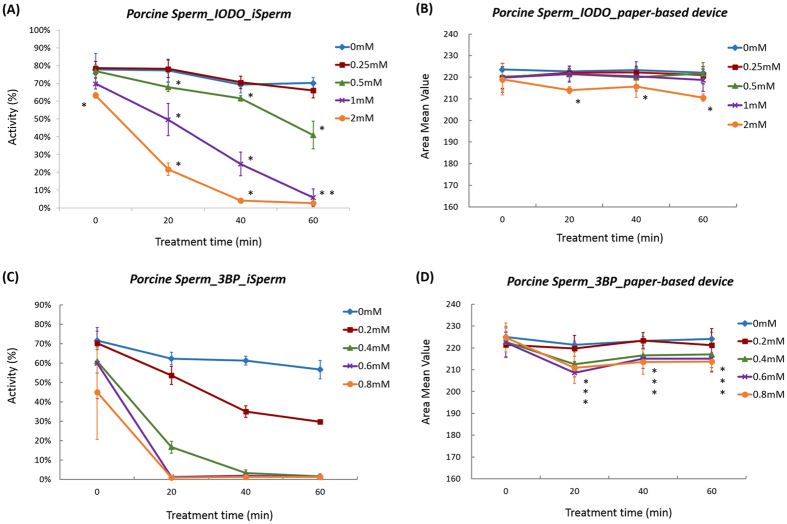
Inhibitor dose-dependent effects of IODO and 3BP. (**A**) iSperm and (**B**) AMV from our paper-based MTT assay following treatment with 0, 0.25, 0.5, 1, and 2 mM of IODO at 37.5 °C. (**C**) motility of iSperm and (**D**) AMVof 3BP-treated and untreated samples. 3BP concentration was 0, 0.2, 0.4, 0.6, or 0.8 mM in this experiment. Error bars are standard deviation (SD) of three samples in each point (N = 3). Asterisks show the difference with the value of control group without inhibitor (P < 0.05).

iSperm and paper-based MTT assay results indicate that IODO and 3BP more strongly inhibited sperm motility and reduced AMV than other inhibitors. We investigated the dose-dependent effects of both IODO and 3BP and summarized our results in Fig. 3. When we exposed porcine sperm to 2 mM IODO, we found significant differences in motility between the inhibitor-treated and control samples from the start of detection (0 min), suggesting that this inhibitor reduced sperm motility immediately. Sperm motility and related enzyme function were sensitive to inhibitor, however detection of MTT reduction exhibited a duller response. Based on a comparison between Fig. 3A and B, when sperm motility was more than 50%, we could not find a difference in MTT reduction using our paper-based device. Soon after inhibitor treatments, decrease of MTT reduction was very small at 0 and 20 min because accumulated reductants such as NADH were not fully oxidized in sperm. With increasing treatment time, some of the accumulated reductants were oxidized by cell oxidants as sperm motility decreased. Therefore, sperm motility inhibition was observed immediately. However, detection of inhibitor function for MTT reduction requires a longer time interval than the time required for sperm motility decrease evaluation. Applications of 1 and 0.5 mM of IODO produced significant decreases in sperm motility, but only after 20 and 60 min, respectively as shown in Fig. 3A (P < 0.05). AMV, however, was only decreased significantly following 20 min of exposure to 2 mM IODO (P < 0.05) (Fig. 3B). AMV (and GAPDH activity) was not affected by treatment with 1 mM or 0.5 mM IODO. We postulate that our MTT assay may be less sensitive to inhibition response, because other enzymatic reactions still occur in IODO-treated samples.

As shown in Fig. 3C, treatment with 0.2, 0.4, 0.6 and 0.8 mM 3BP significantly inhibited sperm motility after 20, 40 and 60 min (P < 0.05). The AMV of our 0.4, 0.6 and 0.8 mM 3BP-treated samples was lower than the AMV of our control sample after 20, 40, and 60 min of reaction (Fig. 3D). However, the AMV value of 0.2 mM 3BP treated sample was almost the same as the control sample AMV. The trend toward dull AMV response to inhibitor treatment was also observed following 3BP treatment. Sperm motility and MTT reduction were more strongly inhibited using 3BP compared to IODO, because the lowest concentrations of 3BP and IODO to induce sperm motility decrease were 0.2 and 0.5 mM, respectively, as shown in Fig. 3. According to references, IODO only inhibits GAPDH activity, while 3BP inhibits both GAPDH and HKII activities^31^,^33^,^38^,^39^. Schmidt *et al*. reported that 1 mM of IODO inhibited GAPDH activity in cultured astrocytes^40^, while ATP production level in tumor cells following treatment with 0.1 mM of 3BP was decreased to 10% of the sample without inhibition^41^. Functional concentrations of the inhibitors used in this study would be related with the abovementioned astrocyte and tumor results.

### Correlation between sperm motility and AMV in MTT assay

**Figure 4:**
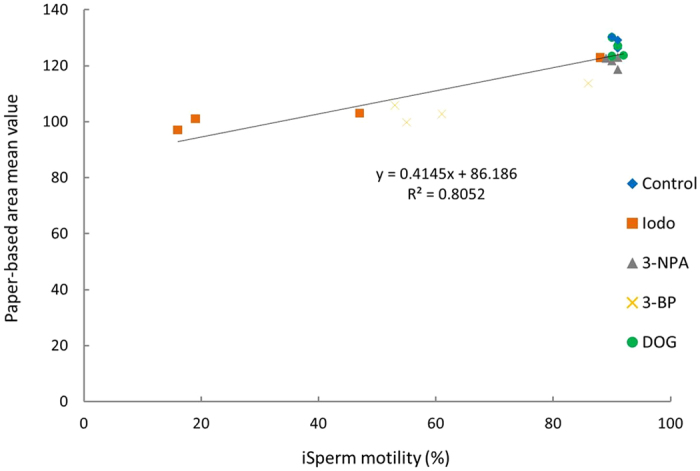
Relationship between motility and difference between AMV in MTT assay (P < 0.05). Data points (N) in this graph are 20.

Figure 4 shows the correlation between sperm motility and AMV in our MTT assay. Data points in Fig. 4 are chosen from averages of different treatment times detailed in Figs 2 and 3. The R^2^ value from this correlation analysis was 0.805 and a significant correlation was found (P < 0.05), suggesting a relationship between these two indicators and a coupling of enzymatic reaction detected by MTT assay with sperm motility. According to literature regarding the relationship between motility and farrowing rate, subfertility cut-off value is 70% motility, and subfertility AMV threshold must be approximately 90% of the original sample based on the regression line parameter calculation^42^. When comparing motility analyses using iSperm and MTT assay results, enzymatic activity and MTT reduction would be higher in semen with greater motility, and semen with higher motility produce more intense (more deeply purple) colorimetric signals.

### Discussion about energy and ATP consumptions for sperm motility

To understand the relationship between sperm motility and MTT reduction inhibition, we first reviewed proposed molecular mechanisms of mammalian sperm motility, which are regulated by motor protein reaction coupled with ATP hydrolysis^43^. In mammalian sperm, ATP can be produced from the catalytic reactions of electron transfer in mitochondria and GAPDH and pyruvate kinase of glycolysis system in the cytosol of flagellum^17^,^33^. The contribution of ATP consumption between these two organelles differs between animal species^17^. In mouse sperm flagellum, an energy transfer reaction scheme is proposed^22^. ATP produced at midpiece mitochondria was diffused to flagella cytosol and was converted to adenosine diphosphate (ADP) by phosphoglycerate kinase (PGK) hydrolysis, and the product 1, 3-bisphosphoglycerate (1, 3-BPG) is catalyzed by GAPDH to produce NADH^22^. By repeating this catalysis by PGK and GAPDH, energy based on ATP produced in mitochondria is transferred to dynein. Therefore, GAPDH works as a key enzymatic reaction in the flagellum for ATP production via glycolysis, energy transfer using ATP from mitochondria, and flagellum beating.

Our experimental results show that porcine sperm motility and MTT reduction were inhibited by the addition of IODO and 3BP. These inhibitors blocked GAPDH catalysis, and reduced sperm motility because of a decrease in ATP production and energy transfer in sperm flagellum. In this study, we did not analyze sperm velocity using CASA in order to discuss the usability of portable diagnosis devices, and we cannot elaborate on sperm velocity changes via inhibition of ATP production. Although sperm motility did not change via inhibition of mitochondrial electron transfer reaction using 3NPA, motile sperm velocity might decrease as a result of decreased total ATP concentration in sperm, according to the report that mentions achievement of higher velocities of mouse sperm in higher respiration/glycolysis ratio^17^. No motility change using DOG in this experiment suggests that glucose uptake is not a critical process for sperm motility enhancement. Our experimental results using these four inhibitor functions support the previous proposals stating that there is an ATP transfer shuttle mechanism from mitochondria to dynein, and that GAPDH is involved as a shuttle pathway enzyme^44^,^45^. Sperm motion energy might be produced by consumption of ATP from both mitochondria and flagellum cytosol. The correlation between sperm motility and mitochondrial enzymatic activity has been found in human sperm^44^. It is likely, therefore, that the suggested ATP shuttle mechanism in porcine sperm might be critical for sperm motility.

### Discussion of MTT reduction in sperm

**Figure 5:**
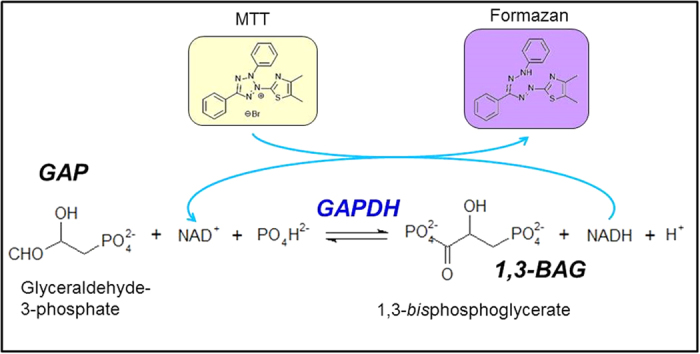
MTT reduction coupled with phosphorylation of GAP catalyzed by GAPDH.

We turned our subsequent attention to the coupling of GADPH catalysis, the mediator function of NADH, and MTT reduction. The relationship between NADH production, GAPDH catalysis, and MTT reduction is depicted in Fig. 5. GAPDH converts glyceraldehyde 3-phosphate to 1,3-bisphosphoglycerate via two-step catalytic reaction, i.e., oxidation of aldehyde to carbonic acid and phosphate transfer^46^,^47^. NADH is synthesized from nicotinamide adenine dinucleotide oxidized form (NAD^+^) via carbonic acid production. Because standard reduction potential of NADH and MTT are −0.32 V and −0.11 V, respectively, NADH with a higher reduction potential can reduce MTT by electron transfer, and NADH is a candidate of the reductants for MTT formazan by direct or indirect electron transfer via mediators to MTT from reductants^47^,^48^,^49^.

The electron transfer would be perturbed by the redox mediators in sperm and semen. MTT reduction occurs at multiple cellular sites in mitochondrial and non-mitochondrial membranes including the endosome/lysosome compartment and the plasma membrane^49^. Small AMV differences in experimental results would be due to other multiple center reactions that are not involved in the signaling cascade for sperm motility. Berg and Nasr-Esfahani *et al*. reported that MTT formazan is accumulated at the midpiece, and recognized as granules under optical microscope^50^,^51^. Redox reactions to reduce MTT catalyzed by redox enzymes occur at midpiece mitochondria and some of these redox reactions would not couple with sperm motility. We consider that the uncoupling of energy transfer reactions to motor protein would be a possible cause of small AMV differences in inhibitor treatment using our paper-based MTT assay in comparison to an optical microscopic motility assay. To find redox enzymes coupled with MTT reduction in sperm cell, detecting the use of mediators, such as phenazine methosulfate based molecules, that facilitate electron transfer to MTT would provide some clues to identifying MTT reducing enzymes in mammalian sperm based on electron transfer kinetics analysis^47^.

### Future Perspective of iSperm and paper-based MTT assays for *in situ* livestock sperm analyses

Our paper-based MTT assay is effective for sperm motility estimation. We show that paper-based MTT can evaluate porcine sperm motility in a manner similar to that reported for evaluating human sperm motility^18^,^19^,^20^. Paper-based assay systems have an advantage over iSperm systems because of the lower initial cost; iSperm systems and optical microscopy detection systems require expensive equipment as shown in Table 2. Another advantage of paper-based assays is the capacity for multiple enzymatic reaction analyses using multiple chemicals in a single assay point. Further, paper-based assay systems can be applied to multiple analytical needs related to sperm enzymatic functions, such as mitochondrial oxygen consumption.

We demonstrated that both portable iSperm and paper-based MTT assay can evaluate porcine sperm motility in resource-limited settings. Although we did not evaluate trajectories of motile sperm compared with a CASA system and sensitivity of paper-based MTT assay was lower than optical spectroscopic system analysis, we could analyze the relationship between sperm motility and enzymatic reactivity using our portable analytical system. Sperm motility mechanism can be discussed based on the results as shown in Fig. 5. Combination assay systems using portable-devices such as iSperm and paper-based MTT can be used in the field to double-check sperm motility analysis when sperm samples cannot be transferred to a resource-rich laboratory.

## Conclusion

Based on the results of our combination assay comparing microscopic observation using iSperm and enzymatic reaction evaluations to inhibitors using our paper-based MTT assay, we demonstrate a relationship between sperm motility, MTT reduction activity, and redox enzymes in porcine sperm. MTT reduction is coupled with NADH production by GAPDH catalysis as demonstrated by use of GAPDH inhibitors IODO and 3BP. Our paper-based MTT assay also could be used to demonstrate energy transfer via redox reactions including GAPDH in sperm flagellum, which is related to sperm motility. According to our results, our paper-based MTT assay can be used for rough estimation of sperm motility in resource-limited settings such as a livestock breeding farm.

## Additional Information

**How to cite this article:** Matsuura, K. *et al*. Relationship between Porcine Sperm Motility and Sperm Enzymatic Activity using Paper-based Devices. *Sci. Rep.* **7**, 46213; doi: 10.1038/srep46213 (2017).

**Publisher's note:** Springer Nature remains neutral with regard to jurisdictional claims in published maps and institutional affiliations.

## Supplementary Material

Supplementary Information

## Figures and Tables

**Table 1:** Inhibitors used in this study.

Chemicals	Maximum concentration	Inhibition
3-nitropropanoic acid (3NPA)	8 mM	Mitochondrial electron transfer between NADH dehydrogenase(I) and ubiquinone
2-deoxy-D-glucose (DOG)	8 mM	Glucose uptake through plasma membrane glucose transporters
iodoacetamide (IODO)	2 mM	Glycolysis (Glyceraldehyde 3-phosphate dehydrogenase (GAPDH))
3-bromopyruvic acid (3BP)	0.8 mM	Both mitochondrial electron transfer (complex II) and glycolysis (HK II, GAPDH)

**Table 2:** Estimated costs for our paper-based device and iSperm in routine sperm motility analyses.

	Cost (USD)	Cost/Assay (USD/assay)
Capital costs		
**Paper**-**based device**		
A smart phone		
withdigital camera	500	
Operating costs		
Plastic straw for sperm application		0.01
Filter paper		0.015
WAX		0.01
MTT		0.01
Total	Capital: 500	Operating: 0.045
**iSperm**		
A smart phone		
withdigital camera	500	
iSperm device	1000	
Software		
Operating costs		
Microchip supplies		1
**Total**	Capital: 1500	Operating: 1
