# Supplementary material for: Relationship between Porcine Sperm Motility and Sperm Enzymatic Activity using Paper-based Devices
Source: Sci Rep. 2017 Apr 7;7:46213. doi: 10.1038/srep46213 (PMC5384208; doi:10.1038/srep46213)
Supplement: Supplementary Information [file srep46213-s1.doc]

Relationship between PorcineSperm Motility and Sperm Enzymatic Activity using Paper-based Devices

Koji Matsuura^1†^, Han-Wei Huang^2†^, Ming-Cheng Chen^2^, Yu Chen^3^* and Chao-Min Cheng^2^*

Koji Matsuura

^1^Department of Biomedical Engineering, Faculty of Engineering, Okayama University of Science, 1-1 Ridai-Cho Kita-Ku, Okayama, 700-0005, Japan

Han-Wei Huang, Ming-Cheng Chen, Chao-Min Cheng

^2^Institute of Biomedical Engineering, National Tsing Hua University, Hsinchu 30013, Taiwan

E-mail: [chaomin@mx.nthu.edu.tw](mailto:chaomin@mx.nthu.edu.tw)

Yu Chen

^3^Department of Urology, Chang Gung Memorial Hospital and Chang Gung University, Taoyuan 33302, Taiwan

E-mail: yu.iok2681@gmail.com

†Equally contributed

Keywords:porcine sperm, sperm motility, enzymatically based paper diagnostic devices, GAPDH

**Supporting Information**

**Correlation between sperm motilities evaluated using iSperm and CASA.**

We examineddifferences in averages andstandard deviations (SDs) in sperm motility using iSperm and CASA as shown in Figure S1A and S1B, respectively. Averages of sperm motility recorded using iSperm were approximately 10% lower than those recorded using CASA. Average SDs in motility analyzed using iSperm and CASA were 5% and 1%, respectively. The difference in averages may be the result of larger SD in our iSperm analysis. Correlation between motilities recorded using CASA and iSperm (Fig S1, 2) was evaluated by Pearson’s correlation coefficient and corresponding significance test. This result suggested that the sperm motility trends of control and inhibitor-treated semen were similar (R^2^=0.988, P<0.05). Therefore, we can sufficiently discuss the sperm motility using iSperm in this study.

**
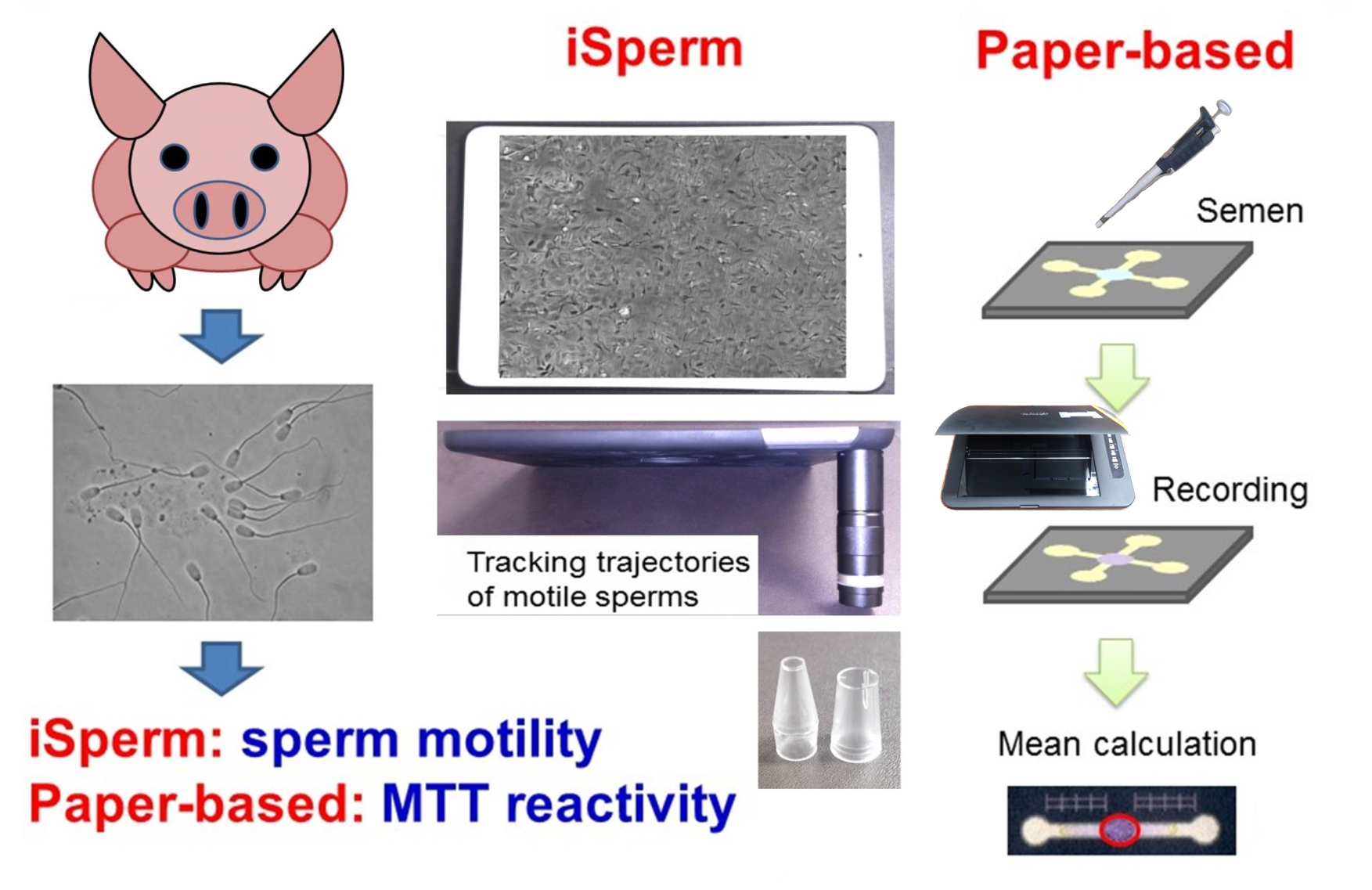
**

**Figure S1.** Overall experimental process schematic.

**
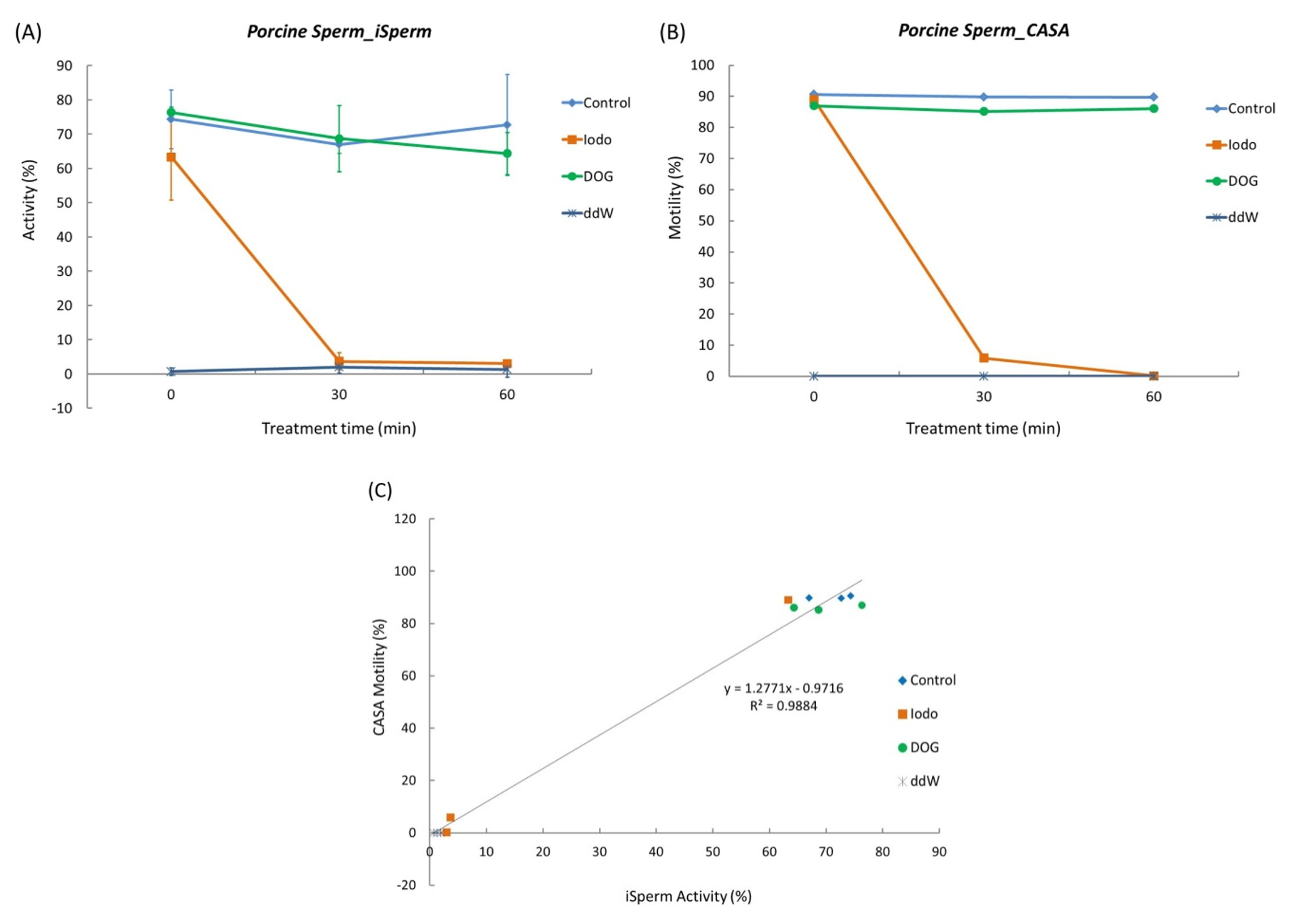
**

**Figure S2.** Comparison of sperm motilities recorded using (A) iSperm and (B) CASA. We used control sample without inhibitor treatment and 2-deoxy-D-glucose (DOG)-treated sample for positive control. iodoacetamide (IODO) was treated for preparation of inhibited sample. Error bars are standard deviation (SD) of three samples in each point (N=3). (C) Correlation between motilities recorded using CASA and iSperm (P<0.05).


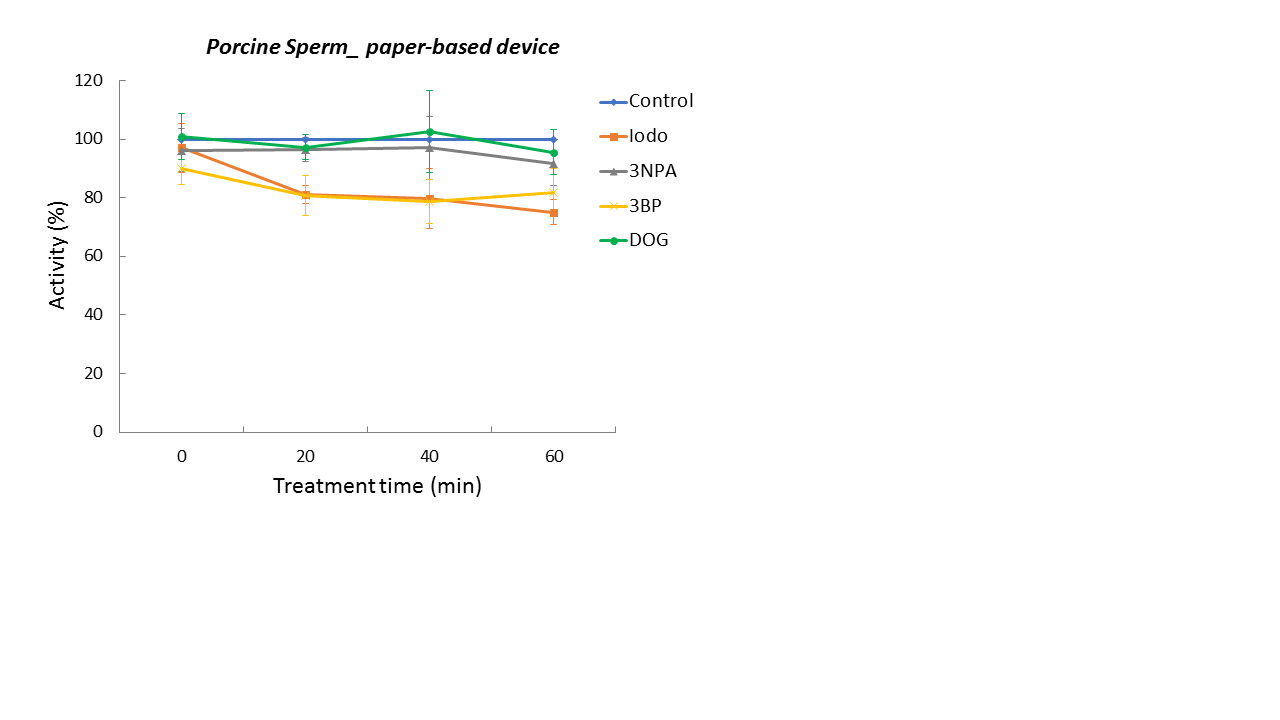


**Figure S3.** AMVs compared to control sample[AMV(X min) / AMV(control at 0min)]*100% (X=20, 40 and 60) in Figure 2.　Error bars are standard deviation (SD) of three samples in each point (N=3).
